# Gu-Ben-Fang-Xiao Decoction Ameliorated Murine Asthma in Remission Stage by Modulating Microbiota-Acetate-Tregs Axis

**DOI:** 10.3389/fphar.2020.00549

**Published:** 2020-05-04

**Authors:** Yingmei Dong, Hua Yan, Xia Zhao, Rui Lin, Lili Lin, Yuanyuan Ding, Liwei Liu, Lishun Ren, Qiongqiong Xing, Jianjian Ji

**Affiliations:** ^1^Affiliated Hospital of Nanjing University of Chinese Medicine, Nanjing, China; ^2^Jiangsu Key Laboratory of Pediatric Respiratory Disease, Institute of Pediatrics, Medical Metabolomics Center, Nanjing University of Chinese Medicine, Nanjing, China; ^3^The First Affiliated Hospital of Zhejiang Chinese Medicine University, Hangzhou, China

**Keywords:** asthma, remission stage, Gu-Ben-Fang-Xiao Decoction, gut microbiota, short-chain fatty acid, regulatory T cell

## Abstract

Dysbiosis of gut microbiota is a critical factor in the pathogenesis of asthma. Manipulating gut microbiota is a promising therapeutic intervention in asthma, and is being extensively studied. Gu-Ben-Fang-Xiao Decoction (GBFXD), derived from traditional Chinese medicine, is an effective and safe therapeutic formula for asthma in remission stage (ARS). Herein, we showed that GBFXD treatment remarkably alleviated ARS by improving respiratory function and lung histopathology. Asthmatic mice displayed a dysbiosis of gut microbiota, represented by significantly increased abundance of *Bacteroidetes* and decreased abundance of *Firmicutes* in gut, while GBFXD treatment reversed the gut dysbiosis in asthmatic mice at phylum, family, and genus levels. Moreover, our data showed that GBFXD treatment increased the abundance of short-chain fatty acid (SCFA)-producing bacteria in asthmatic mice, such as *Firmicutes*, *Lachnospiraceae*, and *Bifidobacteriaceae*, which consequently led to elevated levels of SCFAs. Furthermore, GBFXD treatment significantly enhanced the regulatory T cell differentiation *via* SCFAs, particularly acetate, in asthmatic mice. More critically, the protective effect of GBFXD was shown to be transmissible among asthmatic mice through co-housing microbiota transplantation. Antibiotic cocktail and acetate replenishment experiments also further substantiated the importance of SCFA-producing gut microbiota in GBFXD action. We, thus, demonstrated for the first time that gut microbiota dysbiosis existed in ARS. GBFXD could ameliorate ARS through the microbiota-acetate-Tregs axis.

**Graphical Abstract:**
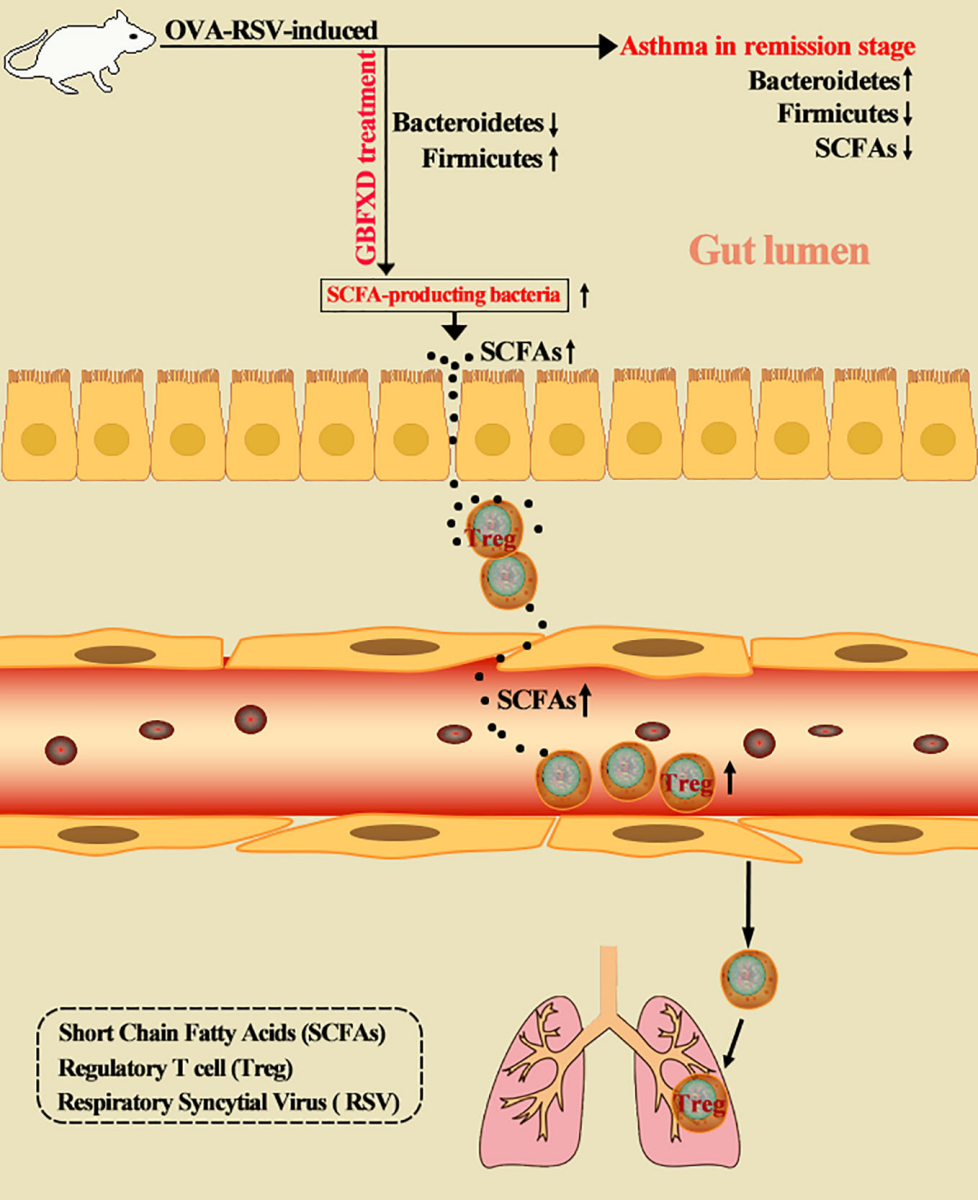


## Introduction

Asthma is a complex heterogeneous disease that is influenced by the interplay of various environmental and genetic factors (Ober and Vercelli, 2011; Krusche et al., 2019). It is one of the most common respiratory diseases affecting more than 334 million individuals (Vos et al., 2012), most of them with childhood-onset (Ferrante and La Grutta, 2018). Since asthma can be triggered by various factors, such as respiratory syncytial virus (RSV) (Shi et al., 2019), allergens, and even physical activities, the predisposing factors are rather challenging to prevent. Moreover, an acute asthma flare-up can be fatal, leaving a heavy burden for families and consuming enormous amounts of economic and medical resources. Therefore, finding effective ways to control the symptoms is necessary for the management of asthma.

In asthma with infrequent symptoms, the symptoms could be better controlled *via* daily maintenance with budesonide than *via* as-needed treatment with budesonide-formoterol (Beasley et al., 2019), highlighting the importance of preventive treatment during asthma remission. However, few related studies were recorded. Currently, inhaled corticosteroids (ICS) and leukotriene receptor antagonists are often prescribed to asthma patients in a clinical remission period (Global Initiative for Asthma, 2019). The moderate use of ICS is effective for early infrequently recurrent asthma or asymptomatic asthma. It can reduce serious asthma-related events and improve lung functions (Papi and Fabbri, 2017; Reddel et al., 2017). However, patients with mild disease often do not adhere to long-course ICS treatments partially due to a fear of adverse effects (Kisa et al., 2003). Besides, young children often find it challenging to use the nebulizer correctly. Even, some patients cannot acquire satisfying clinical outcomes. As a result, a few patients are still under poor drug control. Therefore, novel prevention and treatment strategies for asthma in remission stage (ARS) need to be developed.

The critical role of the gut microbiota in the pathogenesis of asthma was highlighted recently (Ver Heul et al., 2019). Researchers have proposed the concepts of *hygiene hypothesis* (Strachan, 2000) and *gut-lung axis* (Budden et al., 2017), emphasizing that early-life microbiota disruption is likely a predictive index of asthma. Mixed feeding of bacteria from high-risk infants induced asthmatic changes in mice (Arrieta et al., 2015). Suitable gut microbes regulate the host immune system through multiple mechanisms, including direct stimulation of host immunity, and through metabolites, such as short-chain fatty acids (SCFAs) (Trompette et al., 2014), and bile acids (Sánchez, 2018). SCFA is a mediator of *gut-lung axis*, which can boost the production of regulatory T cells (Tregs) (Okada et al., 2010), and thus ameliorate asthma. A clinical study on the microbiome in acute childhood asthma has shown results supporting this phenomenon (Chiu et al., 2019). Manipulating gut microbiota is a promising therapeutic intervention in asthma (Uchiyama et al., 2019). However, no study has explored the role of gut bacteria in ARS.

Gu-Ben-Fang-Xiao Decoction (GBFXD) is an experiential formula proposed by Professor Yuren Jiang, a distinguished pediatrician in Chinese medicine. This formula has been used to treat ARS for decades in China. It has been evidenced as an effective and safe formula for ARS, and GBFXD can significantly reduce asthma recurrence rates and the severity of asthma attacks (Yuan et al., 2010). Our previous studies revealed that GBFXD improved Th1/Th2 immune balance (Liu et al., 2019), lowered endoplasmic reticulum stress (Lu et al., 2016), and downregulated the activity of lipid kinase Sphk1 and the expression of the asthma susceptibility gene ORMDL3 (Huang et al., 2016). The dysbiosis of gut microbiota is a critical factor in the pathogenesis of asthma, but it has not been clarified if gut microbiota was related to the therapeutic benefits of GBFXD. In this study, a mouse model of ARS was established to explore whether gut microbiota was involved in the pathogenesis of ARS and the therapeutic benefits of GBFXD. The levels of SCFAs and the proportions of Treg cells were evaluated to identify the underlying mechanisms. Antibiotic cocktail (ABX) and acetate supplement experiments were applied to explain roles of gut microbiota and acetate on GBFXD action. Our study uncovered for the first time that gut microbiota dysbiosis existed in ARS, and GBFXD could ameliorate ARS through the microbiota-acetate-Tregs axis. This study might provide alternative treatment strategies for ARS.

## Materials and Methods

### Preparation of GBFXD

GBFXD was extracted from 11 components, and the detailed information is shown in **Table 1**. All herbs were purchased from the Affiliated Hospital of Nanjing University of Chinese Medicine and authenticated by Professor Shengjin Liu from Nanjing University of Chinese Medicine. Voucher specimens of *Astragalus mongholicus Bunge* (voucher number: NZY-Zhao-2017001), *Codonopsis pilosula (Franch.) Nannf* (voucher number: NZY-Zhao-2017002), *Atractylodes macrocephala Koidz* (voucher number: NZY-Zhao-2017003), *Poria cocos (Schw.) Wolf* (voucher number: NZY-Zhao-2017004), *Ostrea gigas Thunberg* (voucher number: NZY-Zhao-2017005), *Cryptotympana pustulata Fabricius* (voucher number: NZY-Zhao-2017006), *Citrus × aurantium L* (voucher number: NZY-Zhao-2017007), *Saposhnikovia divaricata (Turcz. ex Ledeb.) Schischk*. (voucher number: NZY-Zhao-2017008), *Magnolia biondii Pamp* (voucher number: NZY-Zhao-2017009), *Schisandra chinensis (Turcz.) Baill* (voucher number: NZY-Zhao-2017010), and *Glycyrrhiza glabra L* (voucher number: NZY-Zhao-2017011) had been deposited in the Herbarium of Traditional Chinese Medicine, Nanjing University of Chinese medicine. GBFXD was decocted, evaporated to a final concentration of 3 g/mL. The quality control information of GBFXD has been previously reported (Xing et al., 2019). Briefly, GBFXD and reference standards solutions were performed by an ultraperformance liquid chromatography (UPLC) (Dionex Ultimate 3000, USA) coupled with LTQ-Orbitrap XL mass spectrometer. Acetonitrile (A) and 0.1% formic acid aqueous solution (B) were selected as the mobile phases. The gradient mobile phase was as follows: 10% to 90% A during 0 to 25 min, 90% A during 26 to 28 min, 90% to 10% A during 29 to 31 min, and 10% A during 32 to 36 min to elute the samples. The oven temperature was 40°C, and the flow rate was 0.3 mL/min. A Hypersil GOLD chromatographic column (100 × 2.1 mm, 1.9 μm, Thermo, USA) was adopted. The system was equipped with an ESI source, and the detection conditions were the same both under positive and negative ion modes. Sheath gas flow rate and auxiliary gas flow rate were 45 arb and 10 arb, respectively. Both the heater temperature and the capillary temperature were 300°C, and the capillary voltage was 35 V. Detailed chemical information of GBFXD was provided in the **Supplementary Figure 1**. Liquiritin, prim-o-glucosylcimifugin, lobetyolin, magnolin, and schisandrol were identified as main bioactive compounds using reference standards (Chengdu Push Bio-technology, China). The results were consistent with our previous research (Lu et al., 2016).

**Table 1:** Components of GBFXD.

Chinese name	Latin name	Family	Part used	Weight (g)	Batch number
Zhi Huang qi	*Astragalus mongholicus Bunge*	Leguminosae	Root	15	140903
Dang shen	Codonopsis pilosula (Franch.) Nannf.	Campanulaceae	Root	10	140901
Bai zhu	Atractylodes macrocephala Koidz.	Composite	Rhizome	10	140903
Fu ling	Poria cocos (Schw.) Wolf.	Polygonaceae	Sclerotia	10	140904
Duan Mu li	Ostrea gigas Thunberg.	Ostreidae	Shell	15	140801
Chan tui	Cryptotympana pustulata Fabricius.	Cicadidae	Slough	6	140406
Chen pi	Citrus × aurantium L.	Rutaceae	Peel	6	140910
Fang feng	*Saposhnikovia divaricata (Turcz. ex Ledeb.) Schischk*.	Umbelliferae	Root	3	140802
Xin yi	Magnolia biondii Pamp.	Magnoliaceae	Bud	6	140428
Wu wei zi	Schisandra chinensis (Turcz.) Baill.	Magnoliaceae	Fruit	6	140901
Gan cao	Glycyrrhiza glabra L.	Leguminosae	Rhizome and root	3	140516

### Mice Treatment

For our work, 4-week-old SPF-grade female BALB/c mice (16-18 g) were purchased from Charles River (Beijing, China). The experimental scheme of the development of the ARS model was described previously (Huang et al., 2016; Lu et al., 2016). Briefly, after a 1-week adaption, mice were sensitized on days 1 and 8 with 100 µg ovalbumin (OVA) and 1 mg AL(OH)_3_ in 0.2 mL NS. From day 15, mice were challenged by aerosolized OVA (2.5% OVA in NS) for 14 consecutive days. On the 29th, 42nd, and 55th days, 10^5^ TCID_50_ RSV was nasally administered to mice under ether anesthesia. From day 32, 2.5% OVA was administered by atomization once every 3 days for eight times. On day 56, mice were divided into four groups, namely, Model, GBFXD, Mont, and dexamethasone (DXM), and individually gavaged with 0.4 mL double distilled water (Model), 24 g/kg GBFXD (GBFXD), 2.6 mg/kg montelukast sodium granules (Merck sharp & Dohme Corp, USA) (Mont), or 1 mg/kg DXM (Shanghai Shangyao Xinyi, China) for four consecutive weeks. Mice as control (CTRL) did not receive any treatments in the first 55 days, and were gavaged with 0.4 mL double distilled water for four consecutive weeks. Mice were anesthetized with 50 mg/kg sodium pentobarbital in NS and sacrificed on day 85 unless otherwise specified.

### Co-Housing Experiment

The treatments of mice in CTRL, Model, and GBFXD were same as described above, and all single-housed. After the last OVA-RSV challenge, half of the Model mice (Co-Model) were Co-housed with mice treated with GBFXD (Co-GBFXD) until the end of the experiment.

### Antibiotic Cocktail (ABX) and Acetate Replenishment Experiments

After the challenges as described above, mice were gavaged daily for 10 days with an antibiotic cocktail (ABX) containing 0.35 mg/mL gentamycin (Fresenius Kabi, China), 5.25 mg/mL kanamycin (JINYIBAI, China), 8500 U colistin (JINYIBAI, China), 2.15 mg/mL metronidazole (JINYIBAI, China), and 0.5 mg/mL vancomycin (Sigma-Aldrich, USA). Afterward, mice in the G-ABX group were administrated with GBFXD daily for 28 days, and a 1:50 dilution of ABX was delivered ad libitum in drinking water (Lei et al., 2016). After the ABX gavage, mice in the G-Ace group were additionally supplemented with 200 mM sodium acetate (Sigma, USA) in the drinking water as described previously (Antunes et al., 2019) along with the GBFXD and diluted ABX treatment same as G-ABX.

### Lung Histopathology

Following serum collection, the middle lobe of the right lung was fixed with 10% paraformaldehyde in PBS. After paraffin embedding, 5-µm sections were stained with hematoxylin and eosin (H&E). The inflammation scores were determined as described previously (Aich et al., 2012; Liu et al., 2019; Xing et al., 2019): 0 represents no inflammation; 1 represents occasional cuffing with inflammatory cells; 2 represents one to two cell layers of inflammatory cells surrounding bronchi or vessels; 3 and 4 represent three to five cell moderate layer, or more than five cell layers of inflammatory cells, respectively. Histopathology was assessed, and inflammation scores were performed in a double-blind manner by two independent researchers.

### Respiratory Function Measurement

On day 85, the respiratory function was measured by whole-body plethysmography (Emka Technologies, Paris, France). Enhanced pause (Penh) was used as an indicator of airway function. Changes in Penhs were assessed with increasing concentrations of aerosolized methacholine (0, 3.125, 6.25, 12.5, 25, 50 mg/mL) (Qiao et al., 2017; Ravanetti et al., 2017).

### Colonic Contents Collection

Scissors and forceps were sterilized by autoclaving and swabbed with 70% ethanol between samples to reduce interference. The sampling method mentioned in the previous study (Guo et al., 2018) was followed. Briefly, 24 hours after the last gavage, 5 cm of the proximal colon was separated and longitudinally dissected. Colon tissue was placed in an Eppendorf tube containing 1 mL sterile PBS and rinsed vigorously. The procedure was repeated once more, and then the colon was discarded. The suspensions from the two tubes were mixed and immediately frozen in liquid nitrogen and stored at −80°C.

### 16S rDNA Sequencing and Analysis

The colonic bacterial DNA was isolated with the E.Z.N.A.^®^ soil kit (Omega Bio-tek, USA) according to the instructions. DNA concentration and purity were investigated by NanoDrop2000. The V3-V4 regions were amplified using specific primers, 338F (5′-ACTCCTACGGGAGGCAGCAG-3′) and 806R (5′-GGACTACHVGGGTWTCTAAT-3′). PCR products were purified with AxyPrep DNA Gel Extraction Kit (Axygen Biosciences, USA). Sequencing of the TruSeq DNA libraries was done on a Miseq PE300 sequencer (Illumina, USA) following the manufacturer’s recommendations. Trimmomatic software was used for quality control and FLASH (Fast Length Adjustment of SHort reads) software was used for splicing. The taxonomic unit was screened for further annotation (Korpela et al., 2016).

### Gas Chromatography-Mass Spectrometry

Fecal SCFAs were quantified using gas chromatography-mass spectrometry (GC-MS) (Trace 1310-TSQ8000 Evo, Thermo, USA) following previous protocols (Shan et al., 2018; Luo et al., 2019). Briefly, gradient concentrations of acetate (ROE, USA), propionate (Dr. Ehrenstorfer, Germany), and butyrate (TCI, China) were prepared for standard curves. 100 mg fecal matter was diluted in 0.005 M NaOH, mixed vigorously, and centrifuged (Allegra 64R; Beckman Coulter, USA) at 13,000 rpm, 4°C for 10 min. The fecal supernatants and standards were mixed with D3-hexanoic acid (Sigma-Aldrich, USA) as an internal standard and then derivatized by mixing with propanol (Belling, China), pyridine (Sigma-Aldrich, USA), propyl chloroformate (Belling, Beijing), and hexane (Sigma-Aldrich, USA).

Then, 1-µL supernatant was absorbed and detected by GC-MS with TG-5MS capillary GC column (0.25 mm × 30 m, 0.25 μm, Thermo), split ratio of 20:1 with 50°C for 0 to 2 min, 50°C to 70°C for 2 to 4 min, 70°C to 85°C for 4 to 9 min, 85°C to 110°C for 9 to 14 min, 110°C to 290°C for 14 to 20 min, and 290°C for 20 to 28 min to separate the substances. A 70-eV EI source was adopted, with 290°C ion transmission line, 230°C ion source, and 1.2 mL/min high purity helium was used as the carrier gas, under full-scan mode; the scan range was 30 to 600 m/z to detect and analyze substances.

### Flow Cytometry

Peripheral blood mononuclear cells (PBMCs) were isolated using the lymphocyte separation medium (TBD, China) according to the manufacturer’s specifications. Cells were blocked using the FcR blocking reagent (Miltenyi Biotec, Germany) and labeled with pre-conjugated anti-CD4, anti-CD25, and anti-Foxp3 antibodies. For intracellular staining, a Foxp3 Fixation/Permeabilization buffer set (Thermo Fisher Scientific, USA) was used. Data were acquired using a BD Accuri™ C6 Plus flow cytometer (BD Biosciences, USA). Populations were gated and analyzed using FlowJo software (Doonan et al., 2019).

### Statistical Analyses

Data are expressed as mean ± SD. GraphPad Prism 7 was used for statistical analyses. Data were analyzed with one-way ANOVA or two-way ANOVA followed by Dunnett’s or Tukey’s *post hoc* tests for multiple comparisons. P < 0.05 was considered statistically significant, and ns means no statistical significance.

## Results

### GBFXD Ameliorated Respiratory Function and Lung Inflammation in OVA-RSV-Induced Murine Model of ARS

**Figure 1:**
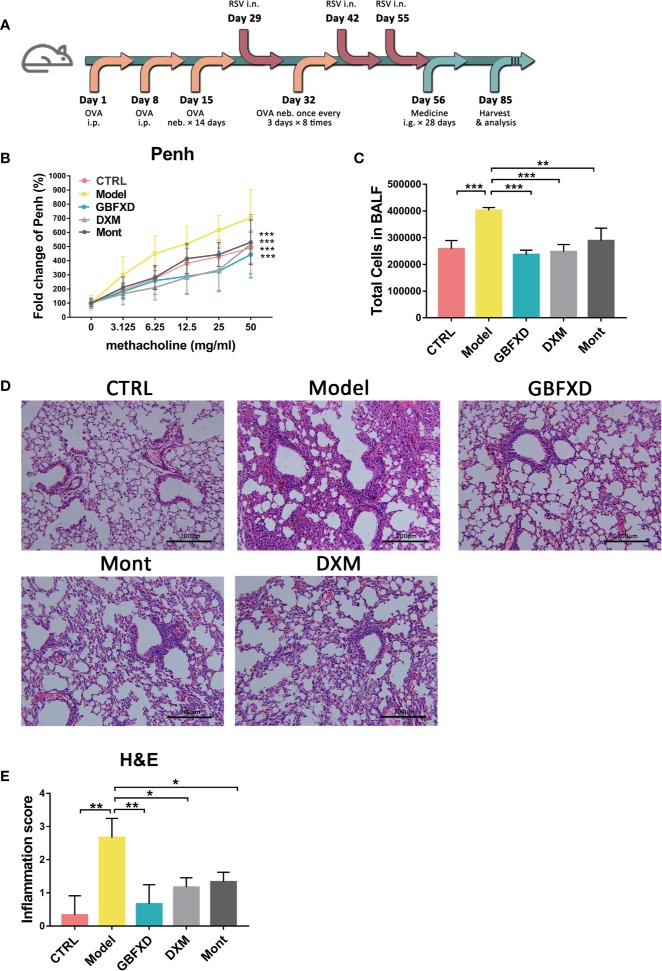
GBFXD ameliorated airway function and lung inflammation in a murine model of ARS. **(A)** Experimental scheme showing the development of the ARS model and the treatments. Ip, intraperitoneal; inh, aerosol inhalation; i.n, nasal administration; ig, intragastric. **(B)** Non-invasive measurement of enhanced pause (Penh) with ascending concentrations of aerosolized methacholine (0, 3.125, 6.25, 12.5, 25, 50 mg/mL) by whole-body plethysmography (WBP). **(C)** Total cell counts in 50 µl BALF using flow cytometry (FCM). **(D)** Representative hematoxylin and eosin (H&E) stained lung sections of mice, 200×. **(E)** Inflammation score of H&E-stained lung sections to estimate perivascular and peribronchiolar inflammation. Data are shown as mean ± SD. Data in **(B)** were analyzed by two-way ANOVA, n = 4 mice per group, data in **(C, E)** were analyzed by one-way ANOVA with Dunnett’s *post hoc* tests for multiple comparisons. n = 3 mice per group, *P < 0.05, **P < 0.01, ***P < 0.001 compared to Model group. ns means no statistical significance.

To mimic the clinical features of ARS, a mouse model was induced by both OVA and RSV (**Figure 1A**), and treated with GBFXD. Airway hyperresponsiveness is a defining feature of asthma. We first examined the Penh of mice to evaluate the effects of GBFXD on airway hyperresponsiveness (**Figure 1B**). Compared with mice in CTRL group, Model group exhibited a significant increase in Penh when challenged with different concentrations of aerosolized methacholine (**Figure 1B**), while GBFXD treatment significantly reversed this progress. The ARS is often accompanied by chronic airway inflammation, characterized by infiltration of inflammatory cells in BALF, and peribronchiolar and perivascular inflammations (**Figures 1C–E**), while GBFXD treatment significantly suppressed infiltration of inflammatory cells in BALF and protected lung injury (**Figures 1C–E**). Collectively, our data showed that GBFXD could ameliorate the pathological conditions and improve the respiratory function in the mouse model of ARS.

### GBFXD Restored the Colonic Microbiota Composition, and Increased the Proportional Proportions of SCFAs-Producing Bacteria

**Figure 2:**
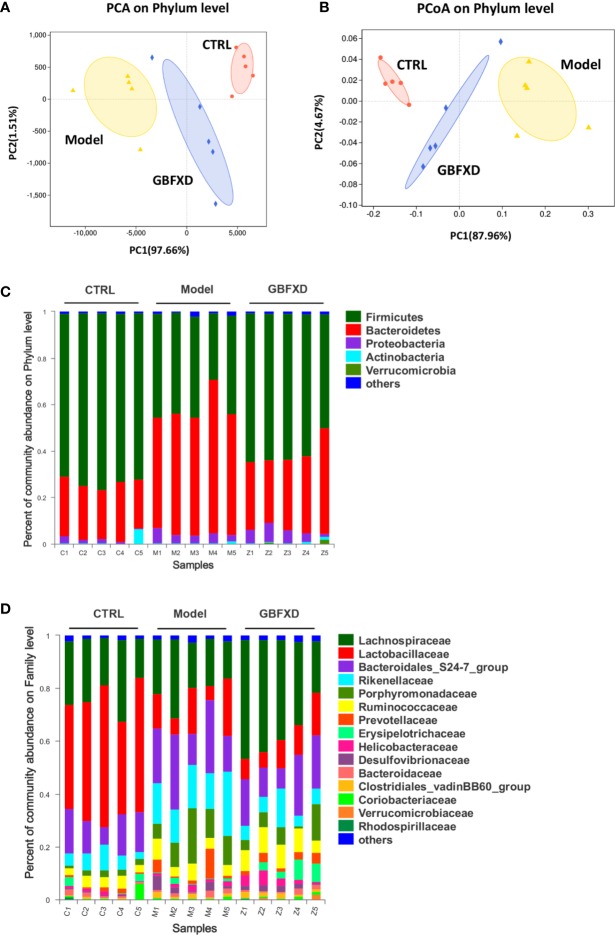
GBFXD reformed mice colonic microbiota composition. **(A**, **B)** Analysis of beta diversity. Principal components analysis (PCA), principal coordination analysis (PCoA), n = 5 mice per group. **(C**, **D)** Detailed relative abundance of community taxa at the phylum and family level within colonic contents determined by 16S rRNA sequencing, n = 5 mice per group (n = 5).

**Figure 3:**
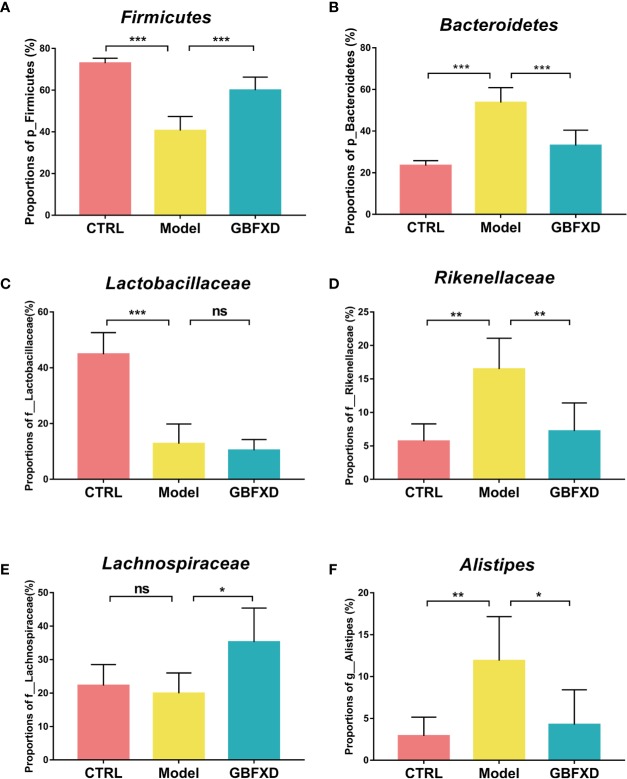
GBFXD treatment reversed the gut dysbiosis in asthmatic mice. **(A**, **B)** Detailed relative abundance of *Firmicutes* and *Bacteroidetes*. **(C–E)** Detailed relative abundance of *Lactobacillaceae*, *Rikenellaceae*, and *Lachnospiraceae*. **(F)** Detailed relative abundance of *Alistipes*. Data are shown as mean ± SD, n = 5 mice per group. Data were analyzed by one-way ANOVA with Dunnett’s *post hoc* tests for multiple comparisons. *P < 0.05,**P < 0.01, ***P < 0.001 compared to Model group, ns means no statistical significance.

Disorders of gut microbiota in acute asthma has been demonstrated previously (Demirci et al., 2019), but whether gut microbiota is involved in ARS is unclear. We thus explored the changes of gut microbiota in ARS, and further investigated whether therapeutic effects of GBFXD on ARS were associated with gut microbiota. The composition of the dominant community exhibited significant changes between CTRL group and Model group, as shown by principal components analysis (PCA) (**Figure 2A**) and principal coordinate analysis (PCoA) (**Figure 2B**). GBFXD partly restored the composition of gut microbiota. In addition, we also performed a compositional analysis of gut microbiota at the phylum (**Figure 2C**), family (**Figure 2D**), and genus levels (**Figure S2A**) respectively. Compared to CTRL group, a decreased proportion of *Firmicutes* (**Figure 3A**) and an increased proportion of *Bacteroidetes* (**Figure 3B**) were observed in the Model group, the imbalance of *Firmicutes* and *Bacteroidetes* was markedly restored by GBFXD treatment (**Figures 3A, B**). At the family level, OVA-RSV induced a decreased abundance of *Lactobacillaceae* (**Figure 3C**) and *f-unclassified-p-Firmicutes* (**Figure S2B**), but the abundance of *Rikenellaceae* (**Figure 3D**), *Porphyromonadaceae*, and *Desulfovibrionaceae* (**Figure S2B**) were significantly increased, while GBFXD treatment reduced the abundance of *Rikenellaceae* (**Figure 3D**), and increased the abundance of *Lachnospiraceae* (**Figure 3E**), *Bifidobacteriaceae*, *Verrucomicrobiaceae*, *Peptococcacea*, *Anaeroplasmataceae*, and *f-unclassified-p-Firmicutes* (**Figure S2C**). At the genus level, *Alistipes* (**Figure 3F**) was more abundant in Model group than in CTRL group, while this bacterium was reduced in GBFXD groups. Altogether, our data indicated ARS mice displayed a dysbiosis of gut microbiota, represented by a significantly increased abundance of *Bacteroidetes* and a decreased abundance of *Firmicutes* in gut, while GBFXD treatment reversed the gut dysbiosis in asthmatic mice at phylum, family, and genus levels.

### GBFXD Effectively Increased the Level of Local and Systemic SCFAs

**Figure 4:**
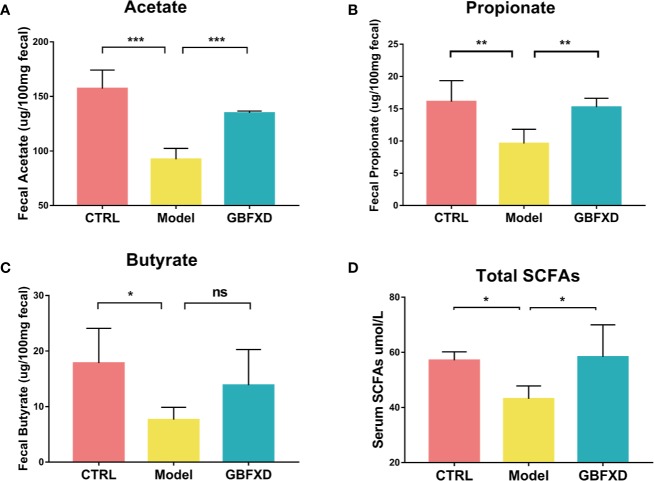
GBFXD increased the levels of total SCFAs in serum, and acetate and propionate levels in the feces. **(A–C)** Acetate, Propionate, and Butyrate concentrations in feces. **(D)** Total SCFAs concentrations in serum. Data are shown as mean ± SD, n = 5 mice per group. Data in **(A–C)** were analyzed by one-way ANOVA with Dunnett’s *post hoc* tests for multiple comparisons. Data in **(D)** were analyzed by Kruskal-Wallis test with Dunn’s multiple comparisons. *P < 0.05, **P < 0.01, ***P < 0.001 compared to Model group. ns means no statistical significance.

Previous studies showed that the ratio of *Firmicutes* to *Bacteroides* (F: B) was generally acknowledged as a predictor of the level of fecal SCFAs (Fernandes et al., 2014), and *Lachnospiraceae* and *Bifidobacteriaceae* were SCFA-producing bacteria (Ji et al., 2019). SCFAs, including acetate, butyrate, and propionate, are immunologically active substances produced by bacteria fermenting carbohydrates in the cecum and colon (Cummings et al., 1987). The increased the ratio of F:B and the abundance of *Lachnospiraceae* and *Bifidobacteriaceae* indicated that the production of SCFAs might be elevated in GBFXD-treated mice. To evidence this hypothesis, GC-MS was utilized to quantify the level of SCFAs in the feces. All levels of SCFAs were significantly decreased in ARS mice (**Figures 4A–C**), particularly the level of acetate, while GBFXD treatment restored the concentrations of acetate and propionate (**Figures 4A, B**). It was reported that part of SCFAs could be accumulated in the circulation, and then cause broad systemic effects (Dodd et al., 2017). In our study, the total level of SCFAs in the blood was significantly reduced in Model group, but GBFXD treatment increased the level of serum SCFAs (**Figure 4D**). Since Acetate accounts for approximately 80% of total SCFAs in the feces (Trompette et al., 2018) and the proportion can be even higher in serum (Thorburn et al., 2015). GBFXD might augment the level of serum acetate. Altogether, our results revealed GBFXD treatment elevated the local colonic and systemic levels of SCFAs.

### Co-Housing With GBFXD-Treated Mice Ameliorated OVA-RSV-Induced Asthma

**Figure 5:**
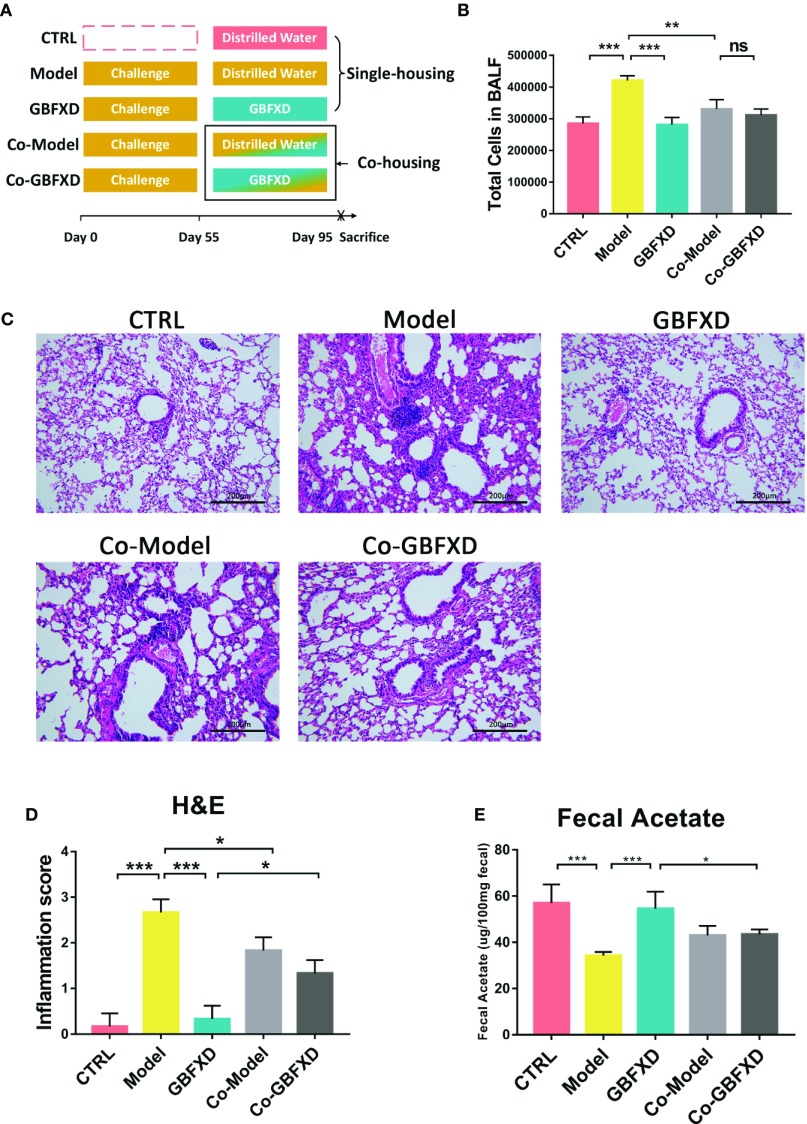
Co-housing ameliorated the inflammatory infiltration in the Model group. **(A)** Experimental scheme of Co-housing experiment. **(B)** Total cell counts in 50 µL BALF determined by flow cytometry (FCM) (n = 3). **(C)** Representative hematoxylin and eosin (H&E)-stained lung sections of mice, 200×. **(D)** Inflammation score of H&E-stained lung sections to estimate perivascular and peribronchiolar inflammation (n = 3). **(E)** Concentrations of acetate in 100-mg feces (n = 5). Data were shown as mean ± SD. Data in **(B, D**, **E)** were analyzed by one-way ANOVA with Tukey’s *post hoc* tests for multiple comparisons. *P < 0.05, **P < 0.01, ***P < 0.001 compared to defined group. ns means no statistical significance.

It is believed that gut microbiota can be transmitted horizontally between mice in the same cage, owing to their coprophagia behavior (Kim et al., 2017). To future explore the role of gut microbiota in GBFXD action, a co-housing experiment was carried out (**Figure 5A**). Total cells in BALF of the Co-Model group were markedly lower than that of the Model mice (**Figure 5B**), while the total cell counts in BALF from Co-GBFXD had a tendency to increase compared to the GBFXD group, but there was no statistical significance (P=0.2023, **Figure 5B**). Consistently, the inflammatory infiltrations surrounding bronchi and vessels in Co-Model group were milder than those in Model mice. However, Co-GBFXD showed more severe peribranchial and perivascular inflammations when compared to the GBFXD group (**Figures 5C, D**). Furthermore, since GBFXD could elevate acetate level, this data suggested that acetate might be responsible for effects of GBFXD on ARS. Thus, the level of acetate was measured in feces of co-housing mice. As shown in **Figure 5E**, the level of acetate in the Co-GBFXD group was significantly reduced than that in GBFXD group, while the level of acetate showed a tendency to increase in Co-Model group, compared to that in Model mice (P=0.1166, **Figure 5E**). Taken together, our results suggested the effects of GBXFD on ARS were dependent on gut microbiota and acetate.

### Microbiota Is Essential for the Prevention of Asthma and the Therapeutic Effects of GBFXD

**Figure 6:**
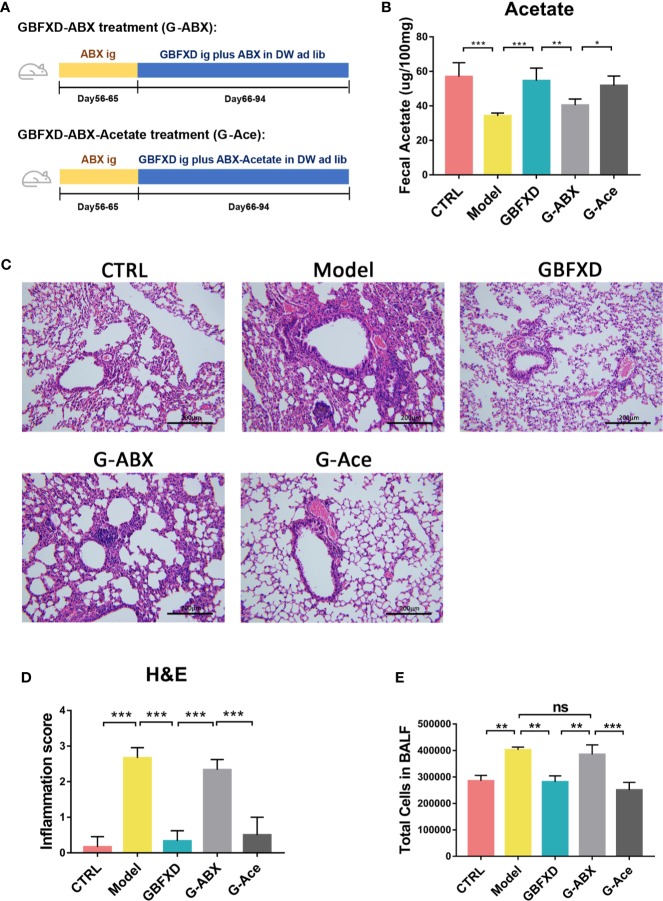
Acetate supplement recovered ABX treatment-induced attenuation of anti-inflammatory effects. **(A)** Experimental scheme of ABX treatment and acetate replenishment experiment. ABX, Antibiotic cocktail; DW, drinking water. **(B)** Total cell counts in 50 µL BALF using flow cytometry (FCM) (n = 3). **(C)** Representative hematoxylin and eosin (H&E) stained lung sections, 200×. **(D)** Inflammation score of H&E-stained lung sections to estimate perivascular and peribronchiolar inflammation (n = 3). **(E)** Concentrations of acetate in feces after interventions (n = 5). Data were shown as mean ± SD. Data in **(B, D, E)** were analyzed by one-way ANOVA with Tukey’s *post hoc* tests for multiple comparisons. *P < 0.05, **P < 0.01, ***P < 0.001 compared to the indicated group. ns means no statistical significance.

To explain the roles of gut microbiota and acetate in therapeutic effects of GBFXD, ABX and acetate replenishment experiments were utilized (**Figure 6A**). ABX was commonly used to disrupt the gut microbiota (Lei et al., 2016). Before treated with GBFXD, mice in G-ABX group were gavaged daily for 10 days with ABX. Afterwards, mice were treated with GBFXD by gavage along with diluted ABX in the drinking water. Mice in the G-Ace group were additionally supplemented with 200 mM sodium acetate in the drinking water. Acetate level in the feces was decreased after ABX administration, and was restored *via* acetate supplement (**Figure 6B**). The protective effects of GBFXD were attenuated after ABX treatment, manifested as augmented histopathological score in the lung section (**Figures 6C, D**) and massive amount of total cells in the BALF (**Figure 6E**), while acetate supplemented restored the therapeutic effects of GBFXD on ARS (**Figures 6C–E**). In conclusion, our results further confirmed that GBFXD ameliorated ARS in a gut microbiota and acetate-dependent manner.

### GBFXD Promoted Treg Cells Differentiation *via* Acetate in ARS Mice

**Figure 7:**
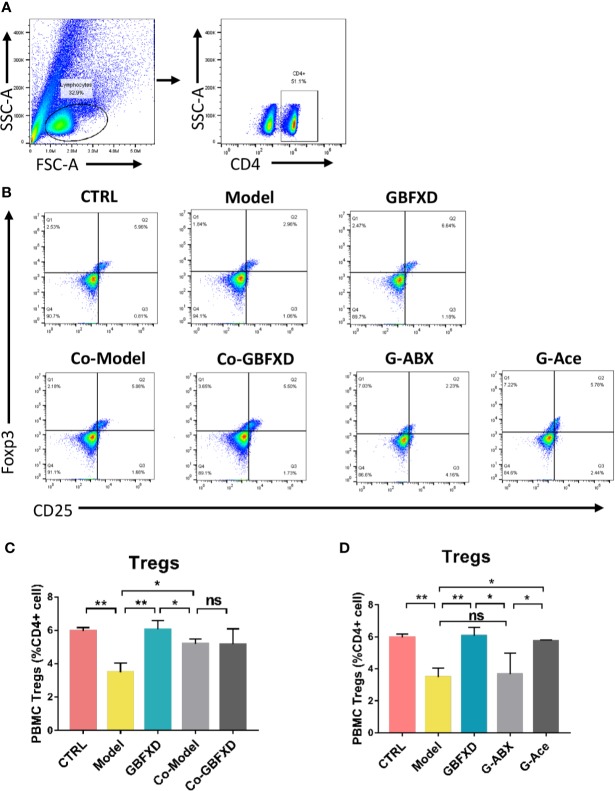
Acetated supplement recovered the ABX-induced reduction of Treg cells. **(A**, **B)** Representative flow cytometry analysis demonstrating Treg cell expression in PBMC subsets. **(C, D)** Treg cell expression in PBMC using FCM. Data were shown as mean ± SD, n = 3 mice per group. Data were analyzed by one-way ANOVA with Tukey’s *post hoc* tests for multiple comparisons. *P < 0.05, **P < 0.01 compared to the indicated group. ns means no statistical significance.

Previous studies have revealed that acetate could promote Foxp3 acetylation and enhance Treg cell numbers and function (Thorburn et al., 2015; Hu et al., 2019). Treg cell is known to downregulate established inflammation in asthma (Lloyd and Hawrylowicz, 2009). The close connection between acetate and Treg cells prompted us to examine whether Treg cell was involved in GBFXD action. Our gating strategy for Treg cell was CD4+CD25+FOXP3+ cell (**Figures 7A, B**). The frequency of Treg cell after GBFXD-treating, co-housing, ABX-treating or acetate-replenishing were examined. The percentage of Treg cells was prominently reduced in Model group, and GBFXD treatment markedly enhanced the proportion of Treg cells. The mice in model group co-housed with GBFXD-treated mice showed an increased percentage of Treg cells in PBMCs (**Figures 7C**), demonstrating that GBFXD could increase the percentage of Treg cells through gut microbiota. Moreover, the effect of GBFXD on Treg cells was abolished after ABX treatment (**Figure 7D**), while acetate supplement restored this effect of GBFXD (**Figure 7D**). This gut microbiota-derived acetate might be responsible for GBFXD induced Treg cell development. In summary, our data indicated that GBFXD treatment significantly enhanced development of Treg cell differentiation *via* gut microbiota-derived acetate. GBFXD might ameliorate ARS through the microbiota-acetate-Tregs axis.

## Discussion

There is no single consistent definition for ARS around the world. Different standards of this concept were applied in different studies, especially for the duration of the asymptomatic period (Burgess et al., 2011; Wang et al., 2019). In China, clinical remission stage of asthma is defined as an asymptomatic state lasting for at least three months (Subspecialty Group of Respiratory Diseases et al., 2016). It is reported that most cases of asthma in clinical remission still show bronchiolar hyperresponsiveness and/or reduced lung function (Guerra, 2005), and chronic inflammation exists even after a five-year symptomless period (Broekema et al., 2010). Considering that most children with asthma are likely to have allergic reactions and history of RSV infection (Sánchez, 2018), we established a murine model induced by OVA and RSV for a better representation of the clinical features. In consistence with the clinical manifestations of ARS, the asthmatic mice in our latest and previous studies (Lu et al., 2016; Liu et al., 2019) exhibited chronic airway inflammation and bronchiolar hyperresponsiveness.

GBFXD was modified from the well-known classic formula Yupingfeng Powder in traditional Chinese medicine (TCM). According to theories from TCM, the lung-spleen qi deficiency constitution is the major pattern for ARS patients (Yun-hong et al., 2019). GBFXD could supplement the lung, solidify the skin, strengthen the spleen and remove the phlegm. It has been used to treat the lung-spleen qi deficiency syndrome in ARS for decades in China. Fermented Yupingfeng polysaccharides, the main composition of GBFXD, could effectively improve the intestinal flora homeostasis (Sun et al., 2016). This study prompted us to examine whether gut microbiota was involved in GBFXD action. To investigate the role of gut microbiota in ARS, in particular, the mucosa-colonization microbiota, we acquired the colonic contents by violently shaking the colon segments in sterile PBS. With the technique development, nowadays researches not only focus on phylum level, but also shift their attention to finer taxonomic levels, including family and genus. More specific bacteria were found to be involved in the pathogenesis of asthma. A comparison of 319 participations in the Canadian Healthy Infant Longitudinal Development Study showed that within 100 days after birth, the relative abundance of the four candidate bacteria of asthma pathogenesis, *Lachnospira, Veillonella, Faecalibacterium*, and *Rothia*, were significantly reduced in infants at risk of asthma, accompanied by a decrease in the concentration of fecal acetate (Arrieta et al., 2015). The reduced abundance of *Lactobacillus* might also enhance the risk of asthma development (Durack et al., 2018). And the *Akkermansia muciniphila* and *Faecalibacterium prausnitzii* levels were reduced in stool samples of acute asthmatic children (Demirci et al., 2019). Furthermore, researches on manipulating gut microbiota to prevent asthma are undergoing. Oral *Bifidobacterium breve MRx0004*, a commensal strain isolated from a healthy person, reduced the immunopathology of peribronchiolar and perivascular, and increased lung CD4+FoxP3+ cells in a murine model of severe asthma (Raftis et al., 2018). In our study, the decreased abundance of *Firmicutes, Lactobacillaceae* might be associated with the pathogenesis of ARS. Although GBFXD did not improve the abundance of *Lactobacillaceae*, it increased the abundance of *Firmicutes, Lachnospiraceae*, and *Bifidobacteriaceae*, which are the primary SCFA-producing bacteria. We speculate that dysbiosis of gut microbiota, particularly decreased SCFA-producing bacteria, may lead to the pathogenesis of asthma. Consequently, manipulating gut microbiota may be a novel alternative treatment strategy for ARS.

The best-studied microbiota metabolites, SCFAs, are produced in the gut and circulate through the hepatic portal vein into the peripheral blood after transepithelial migration (Cummings et al., 1987; Dodd et al., 2017). The circulating SCFAs, including acetate, propionate, and butyrate, have been highlighted as mediators of the gut-lung axis and suppressors of the allergic inflammation (Anand and Mande, 2018; Cait et al., 2018). In our study, GBFXD increased the levels of fecal acetate, propionate, and total serum SCFAs. Since acetate is the most abundant SCFA in the blood, the distinguished change in overall serum SCFAs might ascribe to the acetate concentration. Acetate feeding could enhance the number and function of Treg cells, and thus suppress allergic airway diseases (Thorburn et al., 2015). Co-housing narrowed the differences in SCFAs levels between the GBFXD treated and untreated mice. Due to laboratory limitations, we were unable to use sterile mice to perform strict confirmatory experiments. Since using antibiotics to generate partial germ-free mice is common in exploratory microbiome studies, we utilized antibiotics that were not easily absorbed in the intestine and had broad-spectrum antibacterial effects. The therapeutic effects of GBFXD were attenuated after ABX treatment, while acetate supplemented restored the protective effects of GBFXD, confirming that microbiota and acetate were essential for GBFXD in treating ARS. Further studies are needed to investigate how GBFXD manipulates gut microbiota and microbiota-derived acetate to ameliorate ARS.

Treg cell is a negative regulatory cell that takes part in the regulation of multiple immune responses, its roles in allergy (Larché, 2007), autoimmune, and graft-versus-host reactions have been widely recognized. The roles of Treg cell involve maintaining peripheral tolerance and suppressing inflammation (Lloyd and Hawrylowicz, 2009). When Treg cells are dysfunctional, the failure in suppressing excessive immune responses might lead to the subsequent development and progression of asthma. In this study, we demonstrated that Treg cells were significantly reduced in ARS mice. A significant boost in Treg cells was noted after oral administration of GBFXD. Since co-housing narrowed the differences of Treg cell between the GBFXD-treated and untreated mice, gut microbiota might play an important role in regulating T cell responses. We used ABX to abolish the gut microbiota, and interestingly, the proportion of Treg cells was also decreased, validating the importance of microbiota in Treg cell differentiation. Further experiments showed that by supplementing acetate, the ratio of Treg cells could be restored. These results conclusively indicate that acetate contributes to the modulation of regulatory T cells and induced systemic immune response.

Some differences exist among the current experimental results. For example, a clinical cohort revealed that gut bacterial composition was strongly associated with FEV1 and that the abundance ratio of F: B was higher in adult asthma patients, but the underlying mechanisms were not well studied (Begley et al., 2018). In contrast, the ratio of F: B was lower in ARS mice. The differences between our results and the above adult cohort are possibly due to the differences in species, disease courses, and sampling sites. Further studies are needed both under experimental scenarios and clinical circumstances to find out the critical factors. And it would contribute to revealing the mechanisms underlying the pathogenesis of ARS and help to explore effective therapeutic methods if standardized procedures are established in the future.

## Conclusions

Our data demonstrated for the first time that gut microbiota dysbiosis exists in ARS. GBFXD treatment could ameliorate the pathological conditions of ARS by restoring the dysbiosis of gut microbiota. Further experiments showed that GBFXD could increase the abundance of SCFA-producing bacteria, elevate the local and systemic levels of SCFAs, and enhance Treg cell differentiation where acetate played a crucial role. Regulating microbiota-acetate-Tregs axis may be a promising strategy for treating ARS.

## Data Availability Statement

The datasets generated for this study can be found in the BioProject: PRJNA596640, http://www.ncbi.nlm.nih.gov/bioproject/596640, https://trace.ncbi.nlm.nih.gov/Traces/sra/?study=SRP238183.

## Ethics Statement

All experimental procedures were reviewed and approved by the animal ethics committee of Nanjing University of Chinese medicine, NO. 201809A019, NO. 201901A018, and were in adherence to the Guide for the Care and Use of Laboratory Animals.

## Author Contributions

YDo and HY were responsible for acquisition, analysis, and interpretation of data and had written the first draft of the manuscript, LLin, and LLiu provided information for research design. RL, YDi, LR, and QX participated in the analysis and provided experimental support. XZ and JJ designed this study and edited the final manuscript.

## Funding

This work was partly supported by the National Natural Science Foundation of China (Grant No. 81774367), Postgraduate Research & Practice Innovation Program of Jiangsu Province, China (Grant No. KYCX19_1190), the leading academics training program of Chinese medicine in Jiangsu Province, China (No. SLJ0224), A Project Funded by the Priority Academic Program Development of Jiangsu Higher Education Institutions (PAPD), the Open Projects of the Discipline of Chinese Medicine of Nanjing University of Chinese Medicine Supported by the Subject of Academic priority discipline of Jiangsu Higher Education Institutions (NO.ZYX03KF), and Natural Science Foundation of Jiangsu Province Fund (BK20180825).

## Conflict of Interest

The authors declare that the research was conducted in the absence of any commercial or financial relationships that could be construed as a potential conflict of interest.

## Supplementary Material

Supplementary Figure 1 The chemical profiles of GBFXD using ultra performance liquid chromatography (UPLC). **(A, B)** The total ion chromatogram (TIC) in positive **(A)**, and negative ion mode **(B)**. **(C–G)** The main bioactive compounds of Prim-O-glucosylcimifugin **(C)**, Liquiritin **(D)**, Lobetyolin **(E)**, Magnolin **(F)**, Schisandrol A **(G)**. The bioactive compounds detected in GBFXD were in black peaks and were confirmed by the reference standards in blue peaks.

Supplementary Figure 2 GBFXD reformed mice colonic microbiota composition at the family and genus level. **(A)** Detailed relative abundance of community taxa at the genus level within colonic contents determined by 16S rDNA sequencing. **(B)** Relative abundance of gut microbiota at the family level in CTRL and Model groups. **(C)** Relative abundance of gut microbiota at the family level in GBFXD and Model groups.Data are shown as mean ± SD, n = 5 mice per group. Data in **(B–C)** were analyzed by Wilcoxon rank-sum test. **P* < 0.05.

## Abbreviations

GBFXD, Gu-Ben-Fang-Xiao Decoction; SCFA, short chain fatty acid; Treg, regulatory T cell; PBMC, peripheral blood mononuclear cell; BALF, broncho-alveolar lavage fluid; PCA, principal component analysis; PCoA, principal coordinate analysis; GC-MS, gas chromatography-mass spectrometry; OVA, ovalbumin; RSV, respiratory syncytial virus.
