# Supplementary figures and images for: Gu-Ben-Fang-Xiao Decoction Ameliorated Murine Asthma in Remission Stage by Modulating Microbiota-Acetate-Tregs Axis
Source: Front Pharmacol. 2020 May 4;11:549. doi: 10.3389/fphar.2020.00549 (PMC7212778; doi:10.3389/fphar.2020.00549)

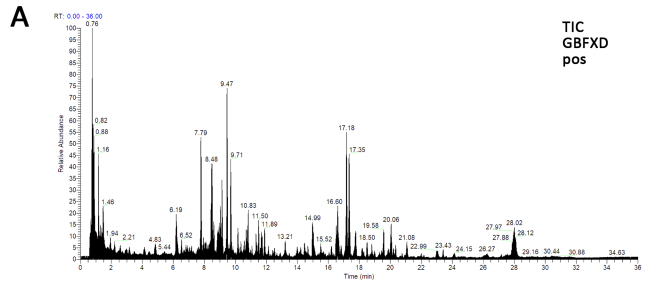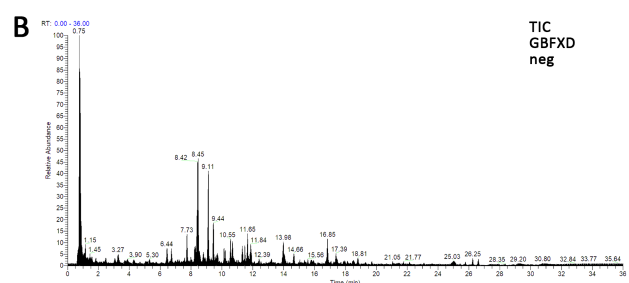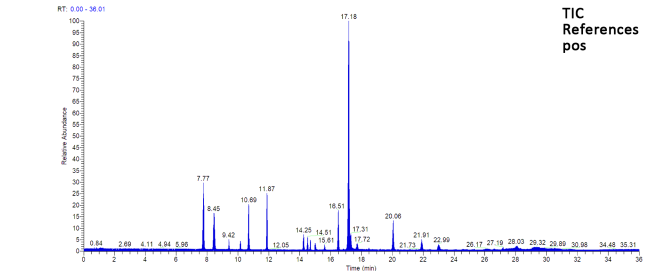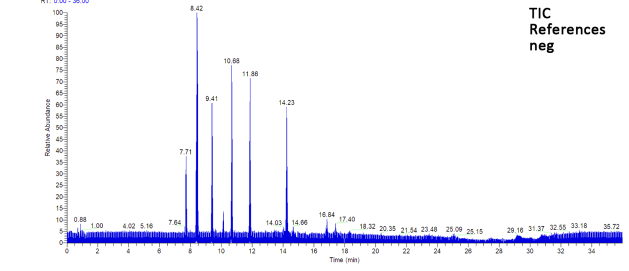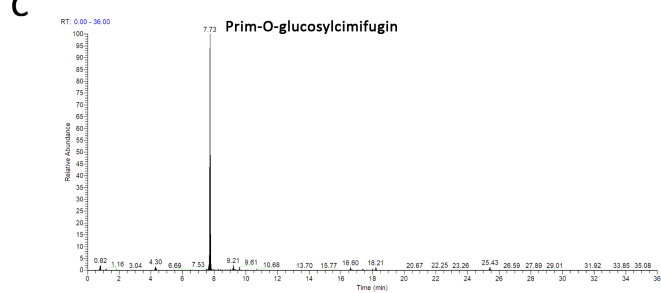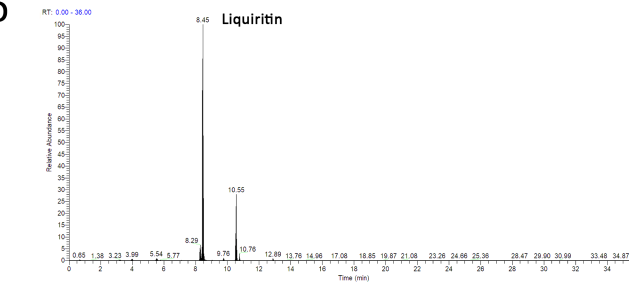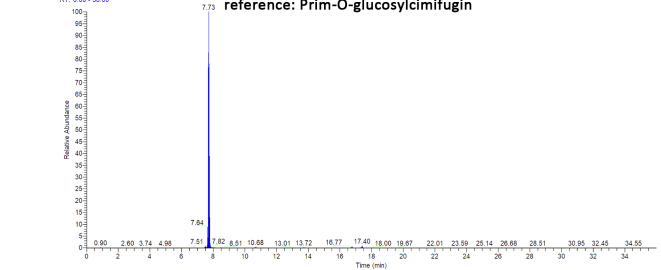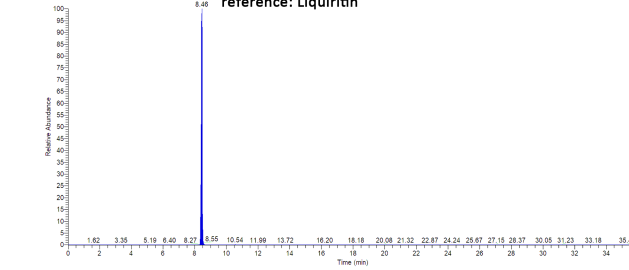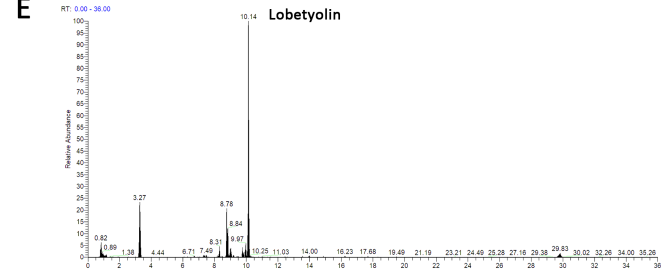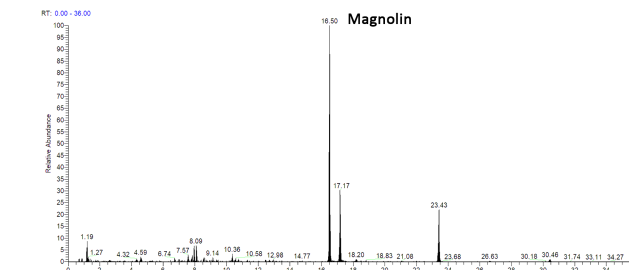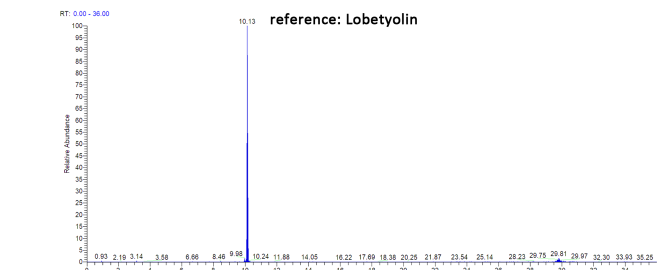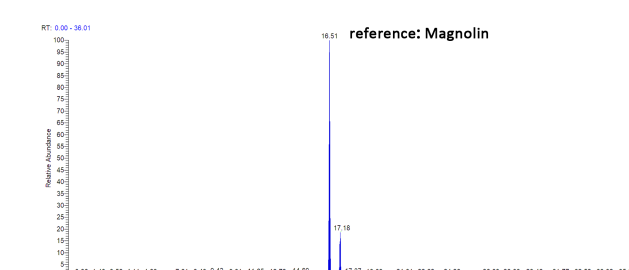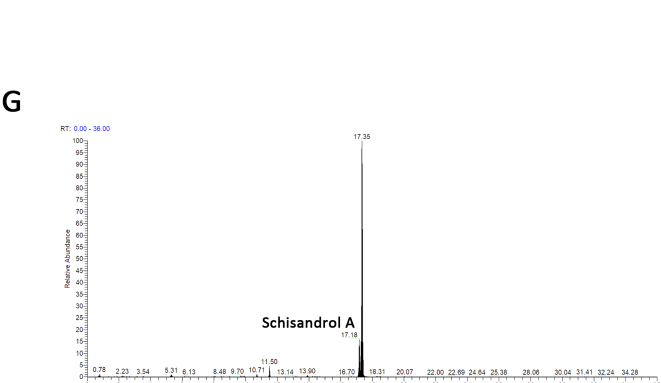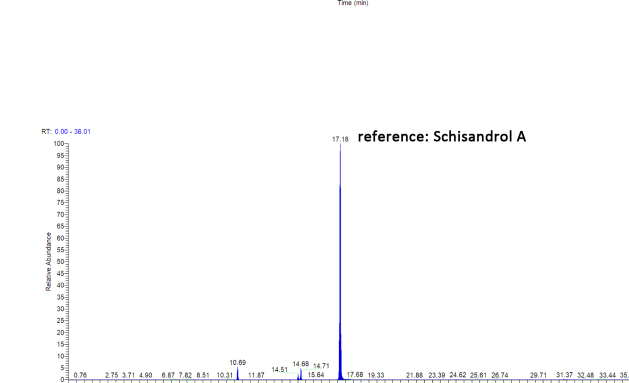

Supplement: Supplementary Figure 1 — The chemical profiles of GBFXD using ultra performance liquid chromatography (UPLC). (A, B) The total ion chromatogram (TIC) in positive (A), and negative ion mode (B). (C–G) The main bioactive compounds of Prim-O-glucosylcimifugin (C), Liquiritin (D), Lobetyolin (E), Magnolin (F), Schisandrol A (G). The bioactive compounds detected in GBFXD were in black peaks and were confirmed by the reference standards in blue peaks. [file Image_1.pdf]
