# Supplementary material for: Gu-Ben-Fang-Xiao Decoction Ameliorated Murine Asthma in Remission Stage by Modulating Microbiota-Acetate-Tregs Axis
Source: Front Pharmacol. 2020 May 4;11:549. doi: 10.3389/fphar.2020.00549 (PMC7212778; doi:10.3389/fphar.2020.00549)
Supplement: Supplementary Figure 2 — GBFXD reformed mice colonic microbiota composition at the family and genus level. (A) Detailed relative abundance of community taxa at the genus level within colonic contents determined by 16S rDNA sequencing. (B) Relative abundance of gut microbiota at the family level in CTRL and Model groups. (C) Relative abundance of gut microbiota at the family level in GBFXD and Model groups.Data are shown as mean ± SD, n = 5 mice per group. Data in (B–C) were analyzed by Wilcoxon rank-sum test. *P < 0.05. [file Image_2.pdf]

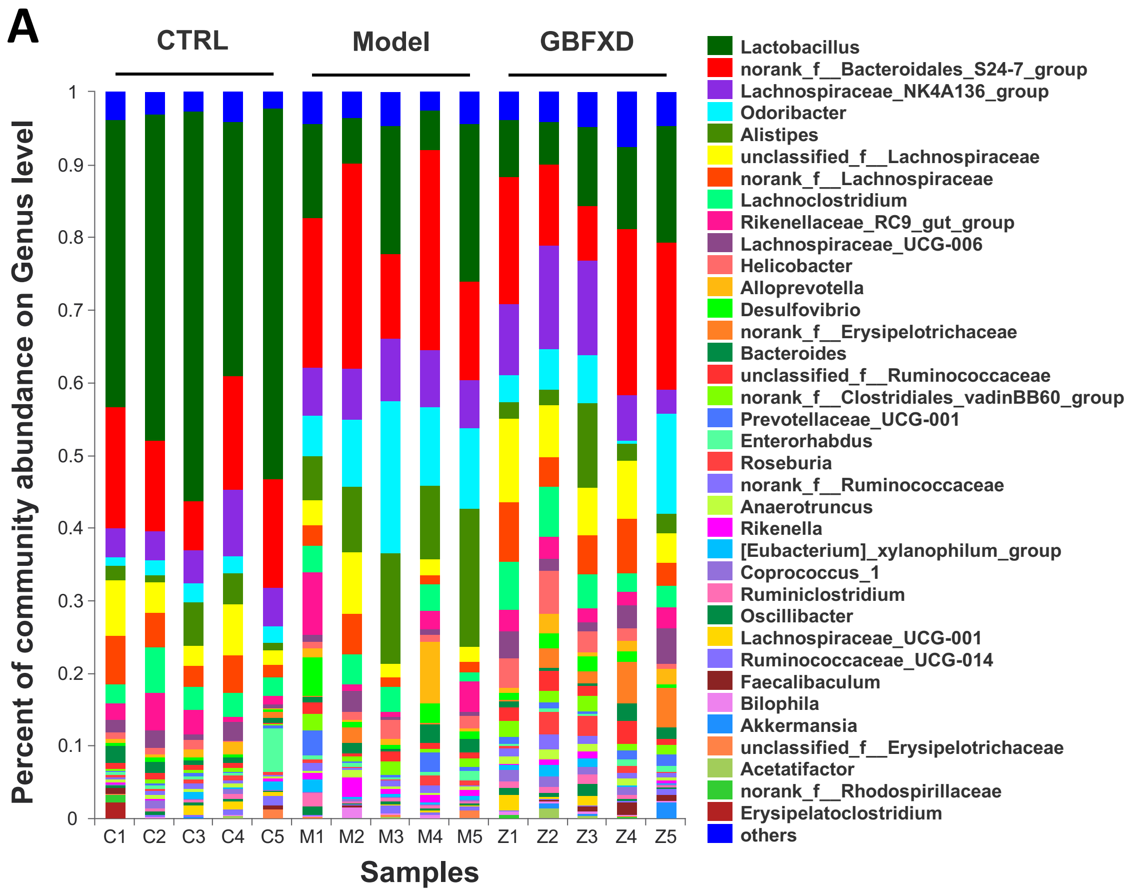

A
CTRL
Model
GBFXD
Percent of community abundance on Genus level
0
0.1
0.2
0.3
0.4
0.5
0.6
0.7
0.8
0.9
1
C1
C2
C3
C4
C5
M1
M2
M3
M4
M5
Z1
Z2
Z3
Z4
Z5
Samples
Lactobacillus
norank_f__Bacteroidales_S24-7_group
Lachnospiraceae_NK4A136_group
Odoribacter
Alistipes
unclassified_f__Lachnospiraceae
norank_f__Lachnospiraceae
Lachnoclostridium
Rikenellaceae_RC9_gut_group
Lachnospiraceae_UCG-006
Helicobacter
Alloprevotella
Desulfovibrio
norank_f__Erysipelotrichaceae
Bacteroides
unclassified_f__Ruminococcaceae
norank_f__Clostridiales_vadinBB60_group
Prevotellaceae_UCG-001
Enterorhabdus
Roseburia
norank_f__Ruminococcaceae
Anaerotruncus
Rikenella
[Eubacterium]_xylanophilum_group
Coprococcus_1
Ruminiclostridium
Oscillibacter
Lachnospiraceae_UCG-001
Ruminococcaceae_UCG-014
Faecalibaculum
Bilophila
Akkermansia
unclassified_f__Erysipelotrichaceae
Acetatifactor
norank_f__Rhodospirillaceae
Erysipelatoclostridium
others

B

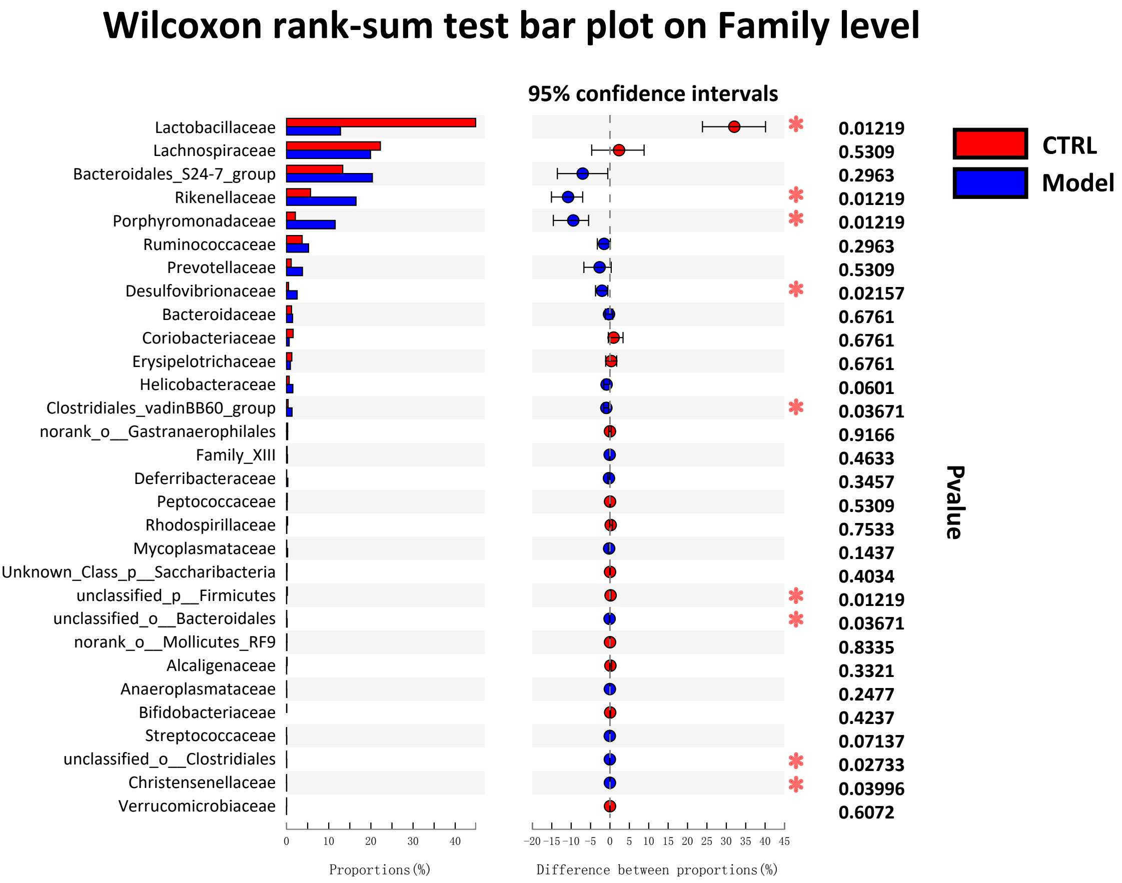
Wilcoxon rank-sum test bar plot on Family level
95% confidence intervals
Lactobacillaceae
Lachnospiraceae
Bacteroidales_S24-7_group
Rikenellaceae
Porphyromonadaceae
Ruminococcaceae
Prevotellaceae
Desulfovibrionaceae
Bacteroidaceae
Coriobacteriaceae
Erysipelotrichaceae
Helicobacteraceae
Clostridiales_vadinBB60_group
norank_o__Gastranaerophilales
Family_XIII
Deferribacteraceae
Peptococcaceae
Rhodospirillaceae
Mycoplasmataceae
Unknown_Class_p__Saccharibacteria
unclassified_p__Firmicutes
unclassified_o__Bacteroidales
norank_o__Mollicutes_RF9
Alcaligenaceae
Anaeroplasmataceae
Bifidobacteriaceae
Streptococcaceae
unclassified_o__Clostridiales
Christensenellaceae
Verrucomicrobiaceae
0.01219
0.5309
0.2963
0.01219
0.01219
0.2963
0.5309
0.02157
0.6761
0.6761
0.6761
0.0601
0.03671
0.9166
0.4633
0.3457
0.5309
0.7533
0.1437
0.4034
0.01219
0.03671
0.8335
0.3321
0.2477
0.4237
0.07137
0.02733
0.03996
0.6072
CTRL
Model
Pvalue
0 10 20 30 40
Proportions (%)
−20 −15 −10 −5 0 5 10 15 20 25 30 35 40 45
Difference between proportions (%)

C

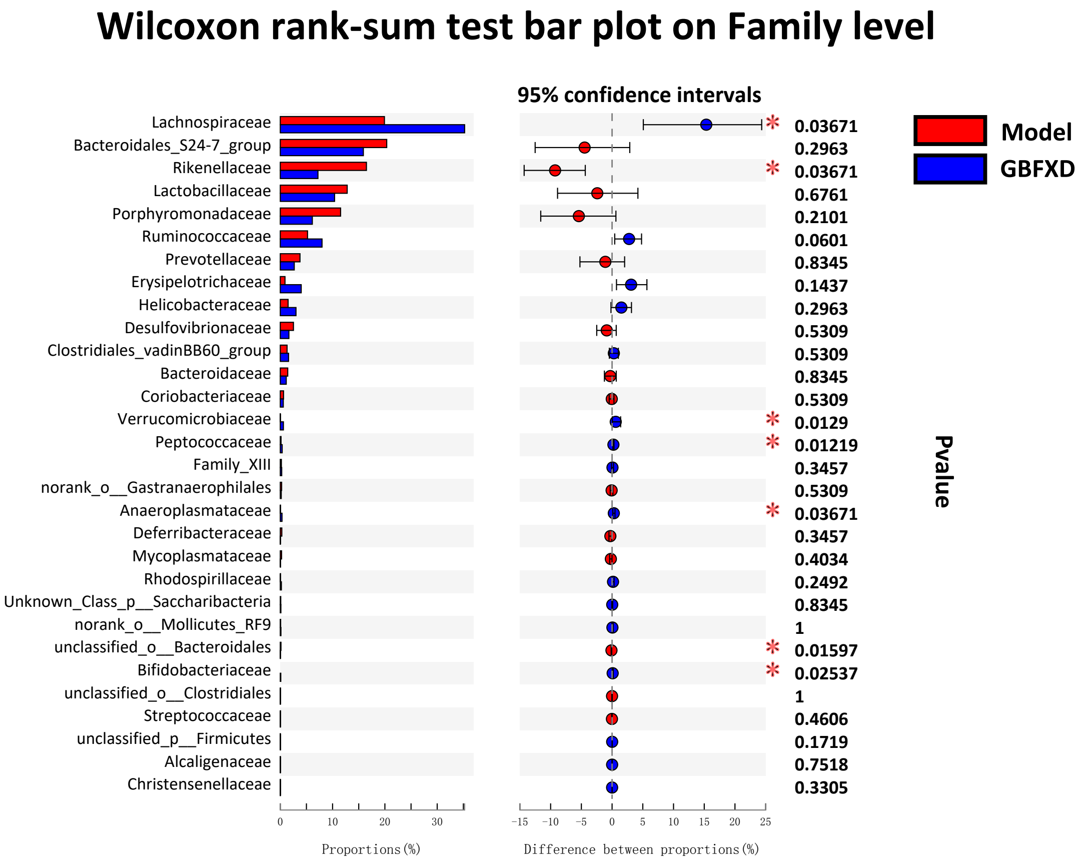
Wilcoxon rank-sum test bar plot on Family level
95% confidence intervals
Lachnospiraceae
Bacteroidales_S24-7_group
Rikenellaceae
Lactobacillaceae
Porphyromonadaceae
Ruminococcaceae
Prevotellaceae
Erysipelotrichaceae
Helicobacteraceae
Desulfovibrionaceae
Clostridiales_vadinBB60_group
Bacteroidaceae
Coriobacteriaceae
Verrucomicrobiaceae
Peptococcaceae
Family_XIII
norank_o__Gastranaerophilales
Anaeroplasmataceae
Deferribacteraceae
Mycoplasmataceae
Rhodospirillaceae
Unknown_Class_p__Saccharibacteria
norank_o__Mollicutes_RF9
unclassified_o__Bacteroidales
Bifidobacteriaceae
unclassified_o__Clostridiales
Streptococcaceae
unclassified_p__Firmicutes
Alcaligenaceae
Christensenellaceae
0.03671
0.2963
0.03671
0.6761
0.2101
0.0601
0.8345
0.1437
0.2963
0.5309
0.5309
0.8345
0.5309
0.0129
0.01219
0.3457
0.5309
0.03671
0.3457
0.4034
0.2492
0.8345
1
0.01597
0.02537
1
0.4606
0.1719
0.7518
0.3305
Model
GBFXD
Pvalue
0 10 20 30
Proportions (%)
−15 −10 −5 0 5 10 15 20 25
Difference between proportions (%)
